# Transcriptome mining, functional characterization, and phylogeny of a large terpene synthase gene family in spruce (*Picea *spp.)

**DOI:** 10.1186/1471-2229-11-43

**Published:** 2011-03-07

**Authors:** Christopher I Keeling, Sabrina Weisshaar, Steven G Ralph, Sharon Jancsik, Britta Hamberger, Harpreet K Dullat, Jörg Bohlmann

**Affiliations:** 1 Michael Smith Laboratories, University of British Columbia, 301-2185 East Mall, Vancouver BC, V6T 1Z4, Canada; 2 Roche Diagnostics Ltd., Forrenstrasse, CH-6343 Rotkreuz, Switzerland; 3 Department of Biology, University of North Dakota, Grand Forks, ND, 58202-9019, USA; 4 Department of Plant Biology and Biotechnology, University of Copenhagen, Thorvaldsensvej 40, opg. 10, 1.-1871 Frederiksberg, Denmark

## Abstract

**Background:**

In conifers, terpene synthases (TPSs) of the gymnosperm-specific TPS-d subfamily form a diverse array of mono-, sesqui-, and diterpenoid compounds, which are components of the oleoresin secretions and volatile emissions. These compounds contribute to defence against herbivores and pathogens and perhaps also protect against abiotic stress.

**Results:**

The availability of extensive transcriptome resources in the form of expressed sequence tags (ESTs) and full-length cDNAs in several spruce (*Picea*) species allowed us to estimate that a conifer genome contains at least 69 unique and transcriptionally active TPS genes. This number is comparable to the number of TPSs found in any of the sequenced and well-annotated angiosperm genomes. We functionally characterized a total of 21 spruce TPSs: 12 from Sitka spruce (*P. sitchensis*), 5 from white spruce (*P. glauca*), and 4 from hybrid white spruce (*P. glauca × P. engelmannii*), which included 15 monoterpene synthases, 4 sesquiterpene synthases, and 2 diterpene synthases.

**Conclusions:**

The functional diversity of these characterized TPSs parallels the diversity of terpenoids found in the oleoresin and volatile emissions of Sitka spruce and provides a context for understanding this chemical diversity at the molecular and mechanistic levels. The comparative characterization of Sitka spruce and Norway spruce diterpene synthases revealed the natural occurrence of TPS sequence variants between closely related spruce species, confirming a previous prediction from site-directed mutagenesis and modelling.

## Background

Conifer trees (order *Coniferales*; Gymnosperms) are extremely long-lived plants that must confront a multitude of biotic and abiotic stresses that vary with the season and over their lifetime. Conifers have evolved several resistance mechanisms that repel, kill, inhibit, or otherwise reduce the success of herbivores and pathogens. These mechanisms include both mechanical and chemical defences that can be present constitutively or that are induced upon challenge [1,2]. As a major part of their constitutive and inducible defensive repertoire, conifers produce an abundant and complex mixture of terpenoids in the form of oleoresin secretions and volatile emissions [2,3]. The diversity of the terpenoids in conifers suggests that, like in other plants [4], an arms race has unfolded in the interactions of conifers with other organisms through the production of specialized (i.e., secondary) metabolites. The diversity of conifer terpenoids includes predominantly monoterpenes, sesquiterpenes and diterpenes, which originate from the activity of a family of terpene synthases (TPSs), and other enzymes, such as cytochromes P450, that may functionalize some of the terpenes [2,5].

Despite much work on individual conifer TPSs [2], the total number of TPSs present in any one conifer species is not yet known since no conifer genome has been sequenced to date. In contrast, the sequenced and annotated genomes of several angiosperm species provide an indication of the diversity of TPSs we might expect to see in any one plant species. For example, the genes encoding putatively active mono-, sesqui-, and di-TPSs number at least 32 in the Arabidopsis (*Arabidopsis thaliana*) genome [6], at least 31 in the rice (*Oryza sativa*) genome [7], at least 32 in the poplar (*Populus trichocarpa*) genome [8], and at least 69 in the genome of a highly inbred grapevine (*Vitis vinifera*) Pinot Noir variety [9,10]. All of these angiosperm genomes contain clusters of duplicated TPS genes. The large genome size of conifers and the diversity of their terpenoid profiles may suggest a similarly sized or potentially larger TPS gene family in conifer species. However, targeted BAC sequencing of a few conifer TPSs from white spruce (*Picea glauca*) did not reveal any genomic clustering of multiple TPS genes in this conifer genome [11,12].

Most of our current knowledge of the size, functional diversity and phylogeny of gymnosperm TPSs is based on targeted cDNA cloning and characterization in two conifer species, grand fir (*Abies grandis*) and Norway spruce (*P. abies*), along with a few TPSs in other gymnosperms [2]. In grand fir, 11 different TPS genes have been functionally characterized [13]. Martin et al. [14] described a set of 9 different TPSs in Norway spruce (*P. abies*) and examined the phylogeny of 29 gymnosperm TPSs, all of which fell into the gymnosperm-specific TPS-d subfamily. A deeper understanding of the diversity and functional complexity of the conifer TPS-d subfamily requires additional gene discovery by transcriptome mining. Large collections of expressed sequence tags (ESTs) and full-length cDNAs (FLcDNAs) exist for several conifer species [15-17] and provide a rich resource for identifying and functionally characterizing new TPSs.

Here, we have analyzed the ESTs and FLcDNAs from Sitka spruce (*P. sitchensis*), white spruce (*P. glauca*), and hybrid white spruce (*P. glauca *× *P. engelmannii*) to identify a comprehensive set of expressed members of the spruce TPS gene family. We have functionally characterized several members from each species for a total of 21 newly characterized spruce TPSs. This work complements previous work in Norway spruce [14] and provides a molecular basis from which to explain much of the chemical complexity of the oleoresin and volatile terpenoids in spruce. Results of the functional gene characterization are discussed in the context of previously reported terpenoid metabolite profiles of oleoresin and volatile emissions in Sitka spruce.

## Results and Discussion

### Identification of unique TPS sequences and isolation of full-length TPS cDNA clones

The *in silico *analysis of 443,665 spruce ESTs identified a total of 506 ESTs corresponding to putative TPSs (Table 1). Assembly of these ESTs into contigs and singlets allowed us to estimate the minimum number of actively expressed TPS genes in each of the three spruce species of our analysis. We identified 69 unique TPS sequences in white spruce, 55 in Sitka spruce, and 20 in hybrid white spruce. Although the rate of gene discovery was dependent on the depth of EST sequencing (Table 1), the substantially deeper EST sequence coverage in white spruce (242,931 ESTs) did not result in a proportional increase of TPS discovery relative to Sitka spruce (174,384 ESTs) and hybrid white spruce (26,350 ESTs), suggesting that the majority of expressed TPSs in the tissues sequenced were captured at the depth of sequencing probed in white spruce and Sitka spruce. The estimate of at least 69 TPSs in white spruce is comparable to the number of putatively active TPS genes found in the sequenced genomes of angiosperms and is perhaps a good approximation of the total number of transcriptionally active TPS genes in a conifer species. From the set of assembled TPS sequences, we examined approximately 170 of the corresponding cDNA clones by restriction digest, colony PCR and/or sequencing to identify those which contained full ORFs. Eighteen FLcDNA clones were selected for subcloning and functional characterization. In addition, three full-length TPS cDNA clones were obtained by RACE cloning or homology-based PCR cloning. As the Treenomix project [16], which generated the available cDNA clones focused its FLcDNA program on Sitka spruce, the majority of the full-length TPS cDNA clones were from this species (12 FLcDNAs). Five full-length TPS cDNA clones originated from white spruce, and four from hybrid white spruce.

**Table 1:** *In silico *identification of TPSs in the EST databases of Sitka spruce, white spruce, and hybrid white spruce

Spruce Species	Total ESTs	Total Singlets Plus Contigs	TPS ESTs*	TPS Singlets	TPS Contigs	Total TPSs
White	242,931	59,449	181	36	33	69
Sitka	174,384	37,533	282	25	30	55
Hybrid White	26,350	13,279	43	10	10	20

*Conifer TPS protein sequences available from NCBI were used to query the three species-specific EST databases using the tBLASTn module of WU-BLAST 2.0 and an E-value cut off of 1 × 10^-5^. The resulting outputs were filtered to exclude duplicates, and then assembled separately by species using CAP3 [49]. The total TPSs represents an estimated minimum number of unique TPSs found in each species.

### Functional characterization of recombinant TPS enzymes

Most previously described conifer TPSs are multi-product enzymes [14,18], and because the identity and relative abundance of TPS products are very sensitive to small changes in amino acid sequence [19-24], it is not possible to accurately predict function based solely upon amino acid sequence similarity/phylogeny. While it might be possible to infer a TPS gene function from the chemical phenotype of a corresponding plant mutant, the genetic resources for such an approach are available only for a very few model systems such as Arabidopsis [25]. Instead, in most systems, the functional annotation of each TPS requires expression and enzyme characterization of recombinant protein.

Recombinant spruce TPSs were expressed in *E. coli *and purified by Ni-affinity chromatography before assaying each individually with geranyl diphosphate (GPP), farnesyl diphosphate (*E*,*E*-FPP), and geranylgeranyl diphosphate (*E*,*E*,*E*-GGPP), the three respective *trans*-prenyl diphosphate substrates of conifer monoterpene synthases, sesquiterpene synthases, and diterpene synthases. Since two recent reports described the occurrence and conversion of *cis*-prenyl diphosphate substrates in tomato [26-28], we also assessed if spruce is likely to produce these additional TPS substrates. Mining of all available spruce EST sequences did not reveal the presence of prenyltransferases for the formation of *cis*-prenyl diphosphate substrates (D. Hall and J. Bohlmann; unpublished results).

**Figure 1:**
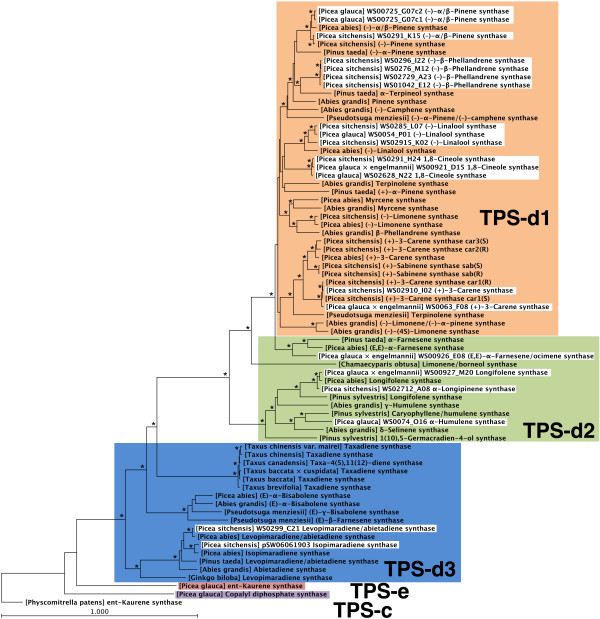
**Phylogeny of functionally characterized gymnosperm TPSs**. The *ent*-kaurene synthase from *Physcomitrella patens *was included as an outgroup. TPSs described in this paper are shown with white background. Protein alignments were prepared using MUSCLE [54] and phylogenetic trees were constructed using the neighbour-joining method with 100 bootstrap repetitions (asterisks are given at clades supported by 80% and higher bootstrap values), within CLC Main Workbench (CLC bio, Århus, Denmark).

**Figure 2:**
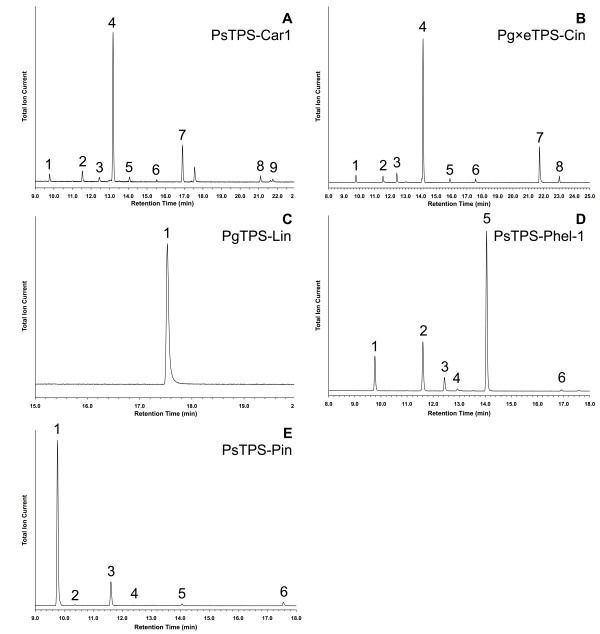
**GCMS total ion chromatogram of products formed by the representative monoterpene synthases PsTPS-Car1, Pg×eTPS-Cin, PgTPS-Lin, PsTPS-Phel-1, and PsTPS-Pin when incubated with GPP**. (A) PsTPS-Car1: 1. (-)-α-pinene, 2. (+)-sabinene, 3. myrcene, 4. (+)-3-carene, 5. β-phellandrene, 6. γ-terpinene, 7. terpinolene, 8. terpinen-4-ol, 9. α-terpineol; (B) Pg×eTPS-Cin: 1. (+)-α-pinene, 2. β-pinene, 3. myrcene, 4. 1,8-cineole, 5. γ-terpinene, 6. unknown, 7. (-)-α-terpineol, 8. unknown; (C) PgTPS-Lin: 1. (-)-linalool; (D) PsTPS-Phel-1: 1. (-)-α-pinene, 2. (-)-β-pinene, 3. myrcene, 4. α-phellandrene, 5. β-phellandrene, 6. terpinolene; (E) PsTPS-Pin: 1. (-)-α-pinene, 2. camphene, 3. (-)-β-pinene, 4. myrcene, 5. β-phellandrene, 6. linalool.

**Figure 3:**
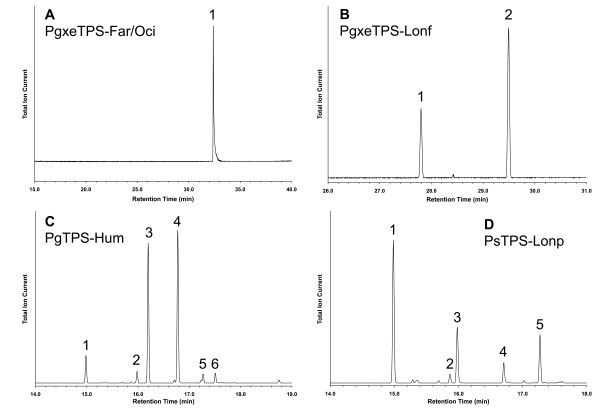
**GCMS total ion chromatogram of products formed by the sesquiterpene synthases Pg×eTPS-Far/Oci, Pg×eTPS-Lonf, PgTPS-Hum, and PsTPS-Lonp when incubated with FPP**. (A) Pg×eTPS-Far/Oci: 1. (*E*,*E*)-α-farnesene; (B) Pg×eTPS-Lonf: 1. α-longipinene, 2. longifolene; (C) PgTPS-Hum: 1. α-longipinene, 2. longifolene, 3. (*E*)-β-caryophyllene, 4. α-humulene, 5. γ-himachalene, 6. α-muurolene; (D) PsTPS-Lonp: 1. α-longipinene, 2. β-longipinene, 3. longifolene, 4. (*E*)-β-farnesene, 5. γ-himachalene.

**Figure 4:**
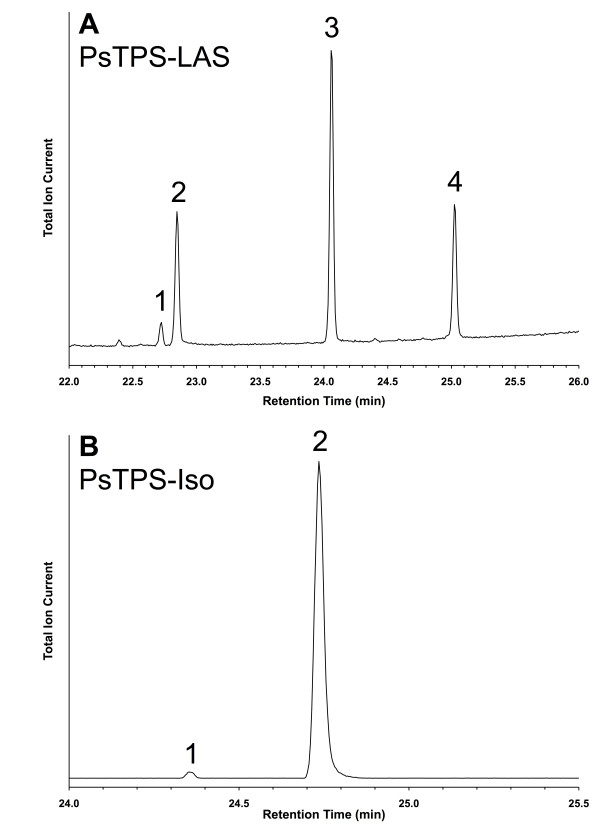
**GCMS total ion chromatogram of products formed by diterpene synthases PsTPS-LAS and PsTPS-Iso when incubated with GGPP**. (A) PsTPS-LAS: 1. palustradiene, 2. levopimaradiene, 3. abietadiene, 4. neoabietadiene; (B) PsTPS-Iso: 1. sandaracopimaradiene, 2. isopimaradiene.

In the following sections we describe the specific functional characterization of the 21 spruce TPSs (Figure 1). With one exception, each of these TPSs only made significant use of one of the substrates. Based upon functional characterization, the 21 TPSs comprised 15 monoterpene synthases, 4 sesquiterpene synthases, and 2 diterpene synthases. The product identities and abundance for each TPS, including quantitative composition of multi-product profiles, is shown in Table 2, and representative GCMS traces are shown in Figures 2, 3, and 4. A summary of the functional annotation along with NCBI GenBank accession numbers appears in Table 3. Results of the functional TPS characterization are discussed in the context of previously reported terpenoid metabolite profiles in Sitka spruce genotype FB3-425 [see Supplemental Tables in [29]], from which many of the functionally characterized TPS FLcDNAs were isolated. Terpenoid profiles are also available from a collection of 111 Sitka spruce accessions [30].

**Table 2:** Product profiles of recombinant TPS enzymes based upon total ion current of GCMS analysis on a DB-WAX column

TPS	Clone ID	Products*	Percent total
MONOTERPENE SYNTHASES			

Pg×eTPS-Car1	WS0063_F08	(+)-3-Carene	53.7
		Terpinolene	17.2
		(+)-Sabinene	5.6
		Terpinen-4-ol	5.2
		(-)-α-Pinene	2.7
		α-Terpineol	2.6
		(-)-β-Phellandrene	2.3
		Myrcene	2.2
		γ-Terpinene	0.9
		α-Terpinene	0.6
		α-Phellandrene	0.3
		α-Thujene	0.2
		Others	6.5
PsTPS-Car1	WS02910_I02	(+)-3-Carene	66.4
		Terpinolene	16.3
		(+)-Sabinene	4.7
		(-)-α-Pinene	2.7
		Terpinen-4-ol	2.5
		(-)-β-Phellandrene	2.1
		Myrcene	2.1
		α-Terpineol	1.4
		γ-Terpinene	0.8
		Others	1.1
PgTPS-Cin	WS02628_N22	1,8-Cineole	89.1
		(-)-α-Terpineol	4.7
		(+)-α-Pinene	1.9
		β-Pinene	1.9
		Unknown	1.4
		Myrcene	1.1
Pg×eTPS-Cin	WS00921_D15	1,8-Cineole	65.6
		(-)-α-Terpineol	18.3
		Myrcene	4.1
		(+)-α-Pinene	3.0
		β-Pinene	2.6
		γ-Terpinene	1.8
		Others	4.6
PsTPS-Cin	WS0291_H24	1,8-Cineole	59.0
		(-)-α-Terpineol	12.2
		Myrcene	9.0
		β-Pinene	5.5
		(+)-α-Pinene	4.7
		Others	9.5
PgTPS-Lin	WS0054_P01	(-)-Linalool	100
PsTPS-Lin-1	WS0285_L07	(-)-Linalool	100
PsTPS-Lin-2	WS02915_K02	(-)-Linalool	100
PsTPS-Phel-1	WS02729_A23	(-)-β-Phellandrene	61.9
		(-)-β-Pinene	18.6
		(-)-α-Pinene	12.3
		Myrcene	5.4
		α-Phellandrene	1.0
		α-Terpinolene	0.5
PsTPS-Phel-2	WS0296_I22	(-)-β-Phellandrene	61.2
		(-)-β-Pinene	19.8
		(-)-α-Pinene	12.1
		Myrcene	5.5
		α-Phellandrene	0.9
		α-Terpinolene	0.5
PsTPS-Phel-3	WS0276_M12	(-)-β-Phellandrene	60.9
		(-)-β-Pinene	20.9
		(-)-α-Pinene	12.5
		Myrcene	4.1
		α-Phellandrene	1.3
		α-Terpinolene	0.2
PsTPS-Phel-4	WS01042_E12	(-)-β-Phellandrene	61.9
		(-)-β-Pinene	19.6
		(-)-α-Pinene	11.5
		Myrcene	5.2
		α-Phellandrene	1.2
		α-Terpinolene	0.6
PgTPS-Pin-1	WS00725_G07c1	(-)-α-Pinene	66.7
		(-)-β-Pinene	33.3
PgTPS-Pin-2	WS00725_G07c2	(-)-β-Pinene	70.5
		(-)-α-Pinene	29.5
PsTPS-Pin	WS0291_K15	(-)-α-Pinene	83.4
		(-)-β-Pinene	12.6
		Linalool	2.1
		β-Phellandrene	1.0
		Camphene	0.4
		Myrcene	0.4

SESQUITERPENE SYNTHASES			

Pg×eTPS-Far/Oci	WS00926_E08	(*E*,*E*)-α-Farnesene/(*E*)-β-ocimene	100
PgTPS-Hum	WS0074_O16	α-Humulene	42.7
		(*E*)-β-Caryophyllene	37.9
		α-Longipinene	7.5
		Longifolene	3.1
		α-Muurolene	2.7
		γ-Himachalene	2.6
		Others	3.4
Pg×eTPS-Lonf	WS00927_M20	Longifolene	69.5
		α-Longipinene	30.5
PsTPS-Lonp	WS02712_A08	α-Longipinene	47.7
		Longifolene	19.9
		γ-Himachalene	15.9
		(*E*)-β-Farnesene	7.0
		β-Longipinene	3.0
		Others	6.4

DITERPENE SYNTHASES			

PsTPS-Iso	pSW06061903	Isopimaradiene	98.3
		Sandaracopimaradiene	1.7
PsTPS-LAS	WS0299_C21	Abietadiene	49.4
		Levopimaradiene	23.8
		Neoabietadiene	23.3
		Palustradiene	3.5

Compounds were identified by comparison of mass spectra and retention indices with authentic standards if available, and retention indices, and/or mass spectra from Adams [52] and NIST, and combined mass spectra and retention index library searches in MassFinder [53] if standards were not available.

**Table 3:** Gene name, origin, accession numbers, and functional annotation of spruce TPS

Gene	Clone ID (genotype)	Functional Annotation*	NCBI Accession
MONOTERPENE SYNTHASES			

Pg×eTPS-Car1	WS0063_F08 (Fa1-1028)	(+)-3-Carene synthase	HQ426152
PsTPS-Car1	WS02910_I02 (FB3-425)	(+)-3-Carene synthase	HQ426167
PgTPS-Cin	WS02628_N22 (PG29)	1,8-Cineole synthase	HQ426160
PgxeTPS-Cin	WS00921_D15 (Fa1-1028)	1,8-Cineole synthase	HQ426156
PsTPS-Cin	WS0291_H24 (FB3-425)	1,8-Cineole synthase	HQ426165
PgTPS-Lin	WS0054_P01 (PG29)	(-)-Linalool synthase	HQ426151
PsTPS-Lin-1	WS0285_L07 (FB3-425)	(-)-Linalool synthase	HQ426164
PsTPS-Lin-2	WS02915_K02 (FB3-425)	(-)-Linalool synthase	HQ426168
PsTPS-Phel-1	WS02729_A23 (FB3-425)	(-)-β-Phellandrene synthase	HQ426162
PsTPS-Phel-2	WS0296_I22 (FB3-425)	(-)-β-Phellandrene synthase	HQ426169
PsTPS-Phel-3	WS0276_M12 (FB3-425)	(-)-β-Phellandrene synthase	HQ426163
PsTPS-Phel-4	WS01042_E12 (Gb2-229)	(-)-β-Phellandrene synthase	HQ426159
PgTPS-Pin-1	WS00725_G07c1 (PG29)	(-)-α/β-Pinene synthase	HQ426153
PgTPS-Pin-2	WS00725_G07c2 (PG29)	(-)-α/β-Pinene synthase	HQ426154
PsTPS-Pin	WS0291_K15 (FB3-425)	(-)-α/β-Pinene synthase	HQ426166

SESQUITERPENE SYNTHASES			

Pg×eTPS-Far/Oci	WS00926_E08 (Fa1-1028)	(*E*,*E*)-α-Farnesene/(*E*)-β-ocimene synthase	HQ426157
PgTPS-Hum	WS0074_O16 (PG29)	α-Humulene synthase	HQ426155
Pg×eTPS-Lonf	WS00927_M20 (Fa1-1028)	Longifolene synthase	HQ426158
PsTPS-Lonp	WS02712_A08 (FB3-425)	α-Longipinene synthase	HQ426161

DITERPENE SYNTHASES			

PsTPS-Iso	pSW06061903 (Haney 898)	Isopimaradiene synthase	HQ426150
PsTPS-LAS	WS0299_C21 (FB3-425)	Levopimaradiene/abieta-diene synthase	HQ426170

*Functional annotation is based on the main terpenoid product(s) of recombinant enzymes expressed in *E. coli*. Most TPSs produced multiple products, as shown in Table 2.

### Functional characterization of monoterpene synthases: (-)-β-phellandrene synthases

We identified four (-)-β-phellandrene synthases in Sitka spruce (PsTPS-Phel-1, PsTPS-Phel-2, PsTPS-Phel-3, and PsTPS-Phel-4), which shared 99% amino acid identity with each other, suggesting that these genes represent nearly identical allelic variants or very recently duplicated genes in the two genotypes that they originated from (Table 3). Interestingly, the Sitka spruce (-)-β-phellandrene synthases were only 70% identical to the (-)-β-phellandrene synthase from grand fir [13]. The phylogenetic distance between the grand fir and Sitka spruce (-)-β-phellandrene synthases (Figure 1) suggests that this specific gene function evolved independently more than once. The identity and approximate quantities of the major [(-)-β-phellandrene, (-)-β-pinene, and (-)-α-pinene] and minor products were nearly identical between the four Sitka spruce (-)-β-phellandrene synthases (Table 2), and the major products and their approximate proportions were also the same between the (-)-β-phellandrene synthases of grand fir and spruce. To the best of our knowledge, a (-)-β-phellandrene synthase has not previously been reported in any other species of spruce [14,31]. In Sitka spruce, β-phellandrene is a major component of the constitutive monoterpene fraction in inner and outer stem tissue and in needles [see Supplemental Tables in [29]]. In stems of Sitka spruce, accumulation of β-phellandrene increased in response to treatment of trees with methyl jasmonate (MeJA) or insect attack [29]. The Sitka spruce (-)-β-phellandrene synthases identified here are likely responsible for this major monoterpenoid component of Sitka spruce oleoresin.

### Functional characterization of monoterpene synthases: (-)-α/β-pinene synthases

We characterized one new (-)-α/β-pinene synthase in Sitka spruce (PsTPS-Pin) and two in white spruce (PgTPS-Pin-1 and PgTPS-Pin-2; both originating from the same genotype) (Tables 1 and 2). These three enzymes clustered closely with the two previously characterized (-)-α/β-pinene synthases from Sitka spruce [32] and Norway spruce [14] in the TPS-d1 clade (Figure 1). The topology of this group of five (-)-α/β-pinene synthases suggests that they represent orthologs in the three spruce species of our comparison. The two pairs of (-)-α/β-pinene synthase genes in white spruce and in Sitka spruce may represent recently duplicated genes or allelic variants in each of these two species. The two white spruce enzymes differed in only four amino acids between each other, and the two Sitka spruce enzymes differed in only six amino acids. The white spruce (-)-α/β-pinene synthases were approximately 96% identical with the (-)-α/β-pinene synthase in Norway spruce, and approximately 96% identical with the (-)-α/β-pinene synthases in Sitka spruce. The Sitka spruce (-)-α/β-pinene synthases shared approximately 95% identity with the Norway spruce enzyme. The (-)-α-pinene synthase from loblolly pine (*Pinus taeda*) [33] and the (-)-α/β-pinene synthase from grand fir [34] clustered outside the group of the spruce (-)-α/β-pinene synthases (Figure 1). These pine and grand fir (-)-pinene synthases may be the corresponding orthologs outside of the spruce genus.

The two (-)-α/β-pinene synthases in white spruce (PgTPS-Pin-1 and PgTPS-Pin-2) contained only four amino acid differences: Q/R94, R/G217, S/N221, and E/G599, but showed an opposing pattern in the relative amounts of α- and β-pinene produced by the recombinant enzymes (67:33 and 29:71 α-pinene:β-pinene, respectively, Table 2). Based upon homology modelling with the limonene synthase from *Mentha spicata *as a template [35], we examined whether any of the four different residues were in or near the active site. Only the residue at 599 (corresponding to M572 of the template) was near the active site. Although this residue was not on the surface of the modelled active site, it was directly behind the residues that contribute to the active site surface near the tail of the substrate analogue (approximately 8.5 Å away). One might hypothesize that the E/G599 difference was the origin of the observed product differences between the two white spruce PgTPS-Pin variants. However, this residue is glycine in a previously characterized (-)-α/β-pinene synthase in Sitka spruce [32] and in a previously characterized (-)-α/β-pinene synthase in Norway spruce [14], which all produce different ratios of α/β-pinene. Therefore, the three other amino acid differences further from the active site also contributed to product profile differences.

In contrast to the white spruce enzymes PgTPS-Pin-1 and PgTPS-Pin-2, the newly characterized Sitka spruce PsTPS-Pin enzyme produced a larger proportion of (-)-β-pinene (more than 80%) and lesser amounts of (-)-α-pinene (less than 13%), but also had four additional minor products not observed with the PgTPS-Pin enzymes (Table 2, Figure 2). This product profile was substantially different from that of the second, previously characterized (-)-α/β-pinene synthase from Sitka spruce, which is dominated by (-)-α-pinene (more than 60% of total product) and lesser amounts of (-)-β-pinene (less than 20% of total product) [32]. Of all five spruce (-)-α/β-pinene synthases, the known Norway spruce enzyme shows the greatest product diversity with (-)-β-pinene (57%), (-)-α-pinene (27%), and (-)-β-phellandrene (11%) as dominant products along with five other minor constituents [14]. Similar to the white spruce (-)-α/β-pinene synthase enzymes, the previously characterized and more distantly related (-)-α/β-pinene synthases from grand fir also produces only (-)-α- and (-)-β-pinene (42% and 58%) [34]. The known product profile of loblolly pine (*Pinus taeda*) (-)-pinene synthase is substantially different, with mostly (-)-α-pinene (79%) with lesser amounts of (-)-β-pinene (4%) and additional minor products [33]. These comparisons of product profiles and ratios across a set of orthologous, or likely orthologous, multiproduct (-)-pinene synthases show that overall sequence relatedness is not a good indicator of the specific product profiles and ratios even for closely related TPS enzymes.

The monoterpenes (-)-α-pinene and (-)-β-pinene are prominent resin compounds in Sitka spruce [29,30] and in Norway spruce [36,37]. In Norway spruce, induced accumulation of these compounds in bark tissue of MeJA-treated stems is the result of increased enzyme activity, protein abundance, and transcript levels of (-)-α/β-pinene synthase [38]. Previous work in Sitka spruce also showed strong accumulation of transcripts detected with a (-)-α/β-pinene synthase probe in MeJA- and insect-treated stems, both at the site of insect feeding and some distance away [29].

### Functional characterization of monoterpene synthases: (-)-Linalool synthases

We characterized two new (-)-linalool synthases in Sitka spruce (PsTPS-Lin-1 and PsTPS-Lin-2) and one in white spruce (PgTPS-Lin) (Tables 2 and 3, Figure 2). Within the TPS-d1 clade, the Sitka spruce and white spruce (-)-linalool synthases formed a group of orthologous genes with the previously cloned Norway spruce (-)-linalool synthase (PaTPS-Lin) [14] (Figure 1). All of these monoterpene synthases were single-product enzymes producing exclusively an acyclic monoterpene alcohol. They shared 86 to 98% amino acid sequence identity, with Sitka spruce PsTPS-Lin1 and white spruce PgTPS-Lin being the most closely related. Since the two (-)-linalool synthases from Sitka spruce (91% identity between them) originated from the same genotype (FB3-425; Table 3), they are likely recently duplicated genes.

(-)-Linalool was previously detected as the major volatile emission of MeJA-treated and weevil-attacked Sitka spruce saplings in the genotype FB3-425 [29], similar to the MeJA-induced emission of linalool from Norway spruce [37]. Transcripts detected with a PaTPS-Lin probe were strongly induced in needles of MeJA-treated Sitka spruce [29]. Linalool volatiles are thought to function in indirect defence against herbivores. Apparently, the (-)-linalool emissions in spruce do not originate from the oleoresin reservoirs of severed resin ducts, but from the induced *de novo *biosynthesis in other tissues. The cloning of (-)-linalool synthase genes from Sitka spruce and white spruce makes it possible to investigate, in future work, the localization of these enzymes and the corresponding transcripts in the needles using the methods of laser-assisted tissue microdissection techniques [39] or immunofluorescence localization [40].

### Functional characterization of monoterpene synthases: (+)-3-Carene synthases

We recently identified a small clade of (+)-3-carene synthases and sabinene synthases in two genotypes of Sitka spruce that are resistant [genotype H898; PsTPS-car1(R), PsTPS-car2(R), and PsTPS-sab(R)] or susceptible [genotype Q903; PsTPS-car1(S), PsTPS-car3(S), and PsTPS-sab(S)] to white pine weevil, *Pissodes strobi *[41]. Here, we identified two additional (+)-3-carene synthases, one in a different genotype of Sitka spruce (genotype FB3-425; PsTPS-Car1), and one in hybrid white spruce (Pg×eTPS-Car1) (Tables 2 and 3, Figure 2). These two (+)-3-carene synthases shared approximately 99% amino acid identity to each other, and were likely the orthologues of the (+)-3-carene synthases PsTPS-car1(R) and PsTPS-car1(S) recently described (Figure 1). Their product profiles were also highly similar for all of the major and most of the minor products. These Sitka and hybrid white spruce (+)-3-carene synthase genes were less similar to the previously characterized Norway spruce (+)-3-carene synthase [42]. A (+)-3-carene synthase gene has not yet been characterized for any conifer outside of the genus *Picea*. The (+)-3-carene synthases were multi-product enzymes, producing predominantly (+)-3-carene synthase (approximately 53 to 66%) and terpinolene (approximately 16%), with lesser amounts of (+)-sabinene and several other minor products (Table 2). Despite the similarity of product profiles, the Sitka spruce and hybrid white spruce (+)-3-carene synthase both shared only 84% percent amino acid sequence identity with the Norway spruce TPS. This highlights how even enzymes with fairly divergent primary sequence can share a similar, complex product profile. The two most abundant products of the Sitka spruce (+)-3-carene synthases, the monoterpenes (+)-3-carene and terpinolene, have recently been identified as indicators for resistance against weevils in a particular geographic region of Sitka spruce origin [30]. Substantial variation exists in the levels of (+)-3-carene across the range of Sitka spruce [30]. The cloning of (+)-3-carene synthases from resistant and susceptible Sitka spruce enabled a detailed characterization of the genetic variability and the molecular underpinnings of (+)-3-carene formation in resistant and susceptible genotypes [41]. Previous work in Sitka spruce showed MeJA- and weevil-induced accumulation of transcripts hybridizing to the Norway spruce (+)-3-carene synthase probe [29]. Similarly, (+)-3-carene synthase was very strongly induced at the transcript, protein, and enzyme activity levels in Norway spruce treated with MeJA [38].

### Functional characterization of monoterpene synthases: 1,8-Cineole synthases

In each of the three spruce species studied we identified and characterized a single 1,8-cineole synthase, PgTPS-Cin, Pg×eTPS-Cin, and PsTPS-Cin (Tables 2 and 3, Figure 2). The three enzymes shared approximately 99% sequence identity to each other and form a distinct group in the TPS-d1 clade most closely related to the linalool synthases. The 1,8-cineole synthases and the linalool synthases are among only a few known conifer monoterpene synthases that produce mainly oxygenated monoterpenes instead of olefins. All three 1,8-cineole synthases were multi-product enzymes with the amount of the major 1,8-cineole product varying from approximately 60% of total product for PsTPS-Cin to approximately 90% for PgTPS-Cin. These three spruce enzymes also had similar profiles of minor products (-)-α-terpineol, (+)-α-pinene, β-pinene, myrcene and others (Table 2 and Figure 2). Although 1,8-cineole has been identified as a monoterpenoid component in needles and MeJA-induced volatile emissions of Norway spruce [37], and has recently been shown to inhibit attraction in the field and response of an olfactory receptor neuron to pheromone of a spruce beetle [43], this is the first characterization of gymnosperm TPSs that produce this compound.

### Functional characterization of sesquiterpene synthases

A complex blend of sesquiterpenes is found in minor quantities in the oleoresin of conifers, including Sitka spruce [29] and Norway spruce [37]. Sesquiterpenes are also present in the MeJA-induced volatile emissions of Norway spruce [37] and in the MeJA- and weevil-induced volatile emissions in Sitka spruce [29]. For the three spruce species of our EST analysis, we cloned and functionally characterized four FLcDNAs, PgTPS-Hum, Pg×eTPS-Lonf, PsTPS-Lonp, and Pg×eTPS-Far/Oci, as *bona fide *sesquiterpene synthases (Tables 2 and 3; Figure 3). PgTPS-Hum, Pg×eTPS-Lonf, PsTPS-Lonp only used FPP as substrate and were typical multi-product conifer sesquiterpene synthases such as those first identified in grand fir [18]. In contrast, Pg×eTPS-Far/Oci was active both with GPP and FPP. This enzyme produced only (*E*,*E*)-α-farnesene, when assayed with FPP, and (*E*)-β-ocimene and a small amount of myrcene, when assayed with GPP. The previously characterized (*E*,*E*)-α-farnesene synthases cloned from Norway spruce [14] and loblolly pine [33] did not show this dual substrate utilization [14,33], although it has been observed with apple (*Malus *× *domestica*) (*E*,*E*)-α-farnesene synthase [44]. (*E*,*E*)-α-farnesene is major sesquiterpene component of the MeJA- and weevil-induced volatile emissions of Sitka spruce [29].

PgTPS-Hum produced predominantly α-humulene (approximately 43%) and (*E*)-β-caryophyllene (approximately 38%), along with several minor products, similar to the α-humulene synthase previously characterized in Scots pine (*Pinus sylvestris*) [45]. Pg×eTPS-Lonf produced longifolene (approximately 70%) and α-longipinene (approximately 30%). Unlike the longifolene synthase from Norway spruce [14], this TPS did not produce other minor products. Sitka spruce PsTPS-Lonp produced predominantly α-longipinene (approximately 48%) but also substantial amounts of longifolene, γ-himachalene, and other minor products. Longifolene and α-longipinene were previously found in the resin of untreated and induced Sitka spruce stems [29] and weevil attack caused an increase of these compounds.

PgTPS-Hum, Pg×eTPS-Lonf, PsTPS-Lonp belong to the TPS-d2 clade of the gymnosperm TPS-d subfamily, together with other conifer multi-product sesquiterpene synthases (Figure 1). The hybrid white spruce Pg×eTPS-Far/Oci appeared to be orthologous with farnesene synthases from loblolly pine and Norway spruce in the TPS-d1 clade.

### Functional characterization of diterpene synthases

Two paralogous diterpene synthases, PsTPS-LAS and PsTPS-Iso, were characterized in Sitka spruce (Tables 2 and 3, Figure 4). These TPSs shared 90% identity and they are the orthologues of levopimaradiene/abietadiene synthase (PaTPS-LAS) and isopimaradiene synthase (PaTPS-Iso) from Norway spruce [14] (Figure 1). They belong to the TPS-d3 clade of the gymnosperm TPS-d family. PsTPS-LAS produced a similar multi-product profile as its ortholog in Norway spruce, composed of abietadiene (49%), levopimaradiene (24%), neoabietadiene (23%), and palustradiene (4%). In contrast to the single-product isopimaradiene synthase from Norway spruce [14], Sitka spruce PsTPS-Iso produced minor amounts of sandaracopimaradiene (2%) in addition to isopimaradiene (98%) (Table 2, Figure 4). PsTPS-Iso is the first gymnosperm TPS identified to naturally produce sandaracopimaradiene, albeit in minor amounts. An *ent*-sandaracopimaradiene synthase has been characterized in rice [46].

PsTPS-LAS and PsTPS-Iso play an important role in the overall diterpene resin acid defence systems of Sitka spruce. The six products of the two Sitka spruce diterpene synthases are present as the corresponding diterpene resin acids in the oleoresin of Sitka spruce stem tissues [29]. Accumulation of all of these diterpene resin acids was induced by MeJA treatment or insect attack, along with increased transcript levels detected with the orthologous PaTPS-LAS and PaTPS-Iso probes [29].

The sequences of PsTPS-LAS and PaTPS-LAS differed by only 12 amino acids, and PsTPS-Iso and PaTPS-Iso differed by only 35 amino acids. In a detailed investigation of the PaTPS-LAS and PaTPS-Iso enzymes, using reciprocal site-directed mutagenesis and domain-swapping, we have recently shown that four amino acid residues determine the different product profiles of these Norway spruce diterpene synthases [24]. These product-determining residues are identical between the levopimaradiene/abietadiene synthases (PsTPS-LAS and PaTPS-LAS) in Sitka and Norway spruce, consistent with their similar product profiles. However, only three of these residues are identical between the isopimaradiene synthases (PsTPS-Iso and PaTPS-Iso) in Sitka and Norway spruce; the fourth residue (V732) is the same as that found in the Norway spruce levopimaradiene/abietadiene synthase. In our previous study [24], the corresponding reciprocal L725V mutation obtained by site-directed mutagenesis of PaTPS-Iso resulted in the formation of sandaracopimaradiene as a minor product. This product profile change is consistent with the new observation that the isopimaradiene synthase from Sitka spruce (PsTPS-Iso) naturally produced sandaracopimaradiene as a minor compound (Table 2, Figure 4). Overall, these results highlight how mutations produced in the laboratory that determine product profile differences also exist in nature and do result in the evolution of altered TPS product profiles between species or genotypes.

### Phylogeny of gymnosperm TPSs

All known conifer TPSs of specialized (i.e., secondary) metabolism are members of the gymnosperm-specific TPS-d subfamily, which is a distinct clade of the larger plant TPS gene family [47]. The TPS-d subfamily has been subdivided into three clades TPS-d1 through TPS-d3 based on a previous phylogeny of 29 gymnosperm TPSs [14]. Here, we have substantially expanded the phylogeny of functionally characterized gymnosperm TPSs to a total of 72 members (Figure 1), of which 41 are from spruce species with 20 different TPSs from Sitka spruce. The number of TPSs functionally characterized in Sitka spruce is one of the largest for any species, but is not yet approaching our *in silico *minimum estimate for the number of TPSs in a spruce genome (at least 69 transcriptionally active TPS genes). The diverse set of newly characterized spruce TPSs broadly represent the major TPS-d1, TPS-d2 and TPS-d3 clades, and allowed us to identify groups of likely orthologous TPS genes across the spruce species. Examples for such groups of orthologous TPSs in the TPS-d1 clade are the (-)-α/β-pinene synthases, the (-)-linalool synthases, (*E*,*E*)-α-farnesene synthases; in the TPS-d2 clade are the longifolene synthases; and in the TPS-d3 clade are the levopimaradiene/abietadiene synthases and isopimaradiene synthases. These groups represent genes whose functions had apparently evolved prior to speciation of the spruce genus. In the TPS-d3 group of conifer diterpene synthases, the basal function of a multi-product levopimaradiene/abietadiene synthase had apparently evolved prior to conifer speciation, as this function exists in a group of closely related genes from the genera *Abies*, *Pinus *and *Picea*.

Overall, the large diversity of gene functions among the many closely related genes of the conifer TPS-d1 group illustrates the many events of gene duplications and sub- or neo-functionalizations that have occurred in the evolution of this amazing family of conifer genes of specialized metabolism. The functionally identified spruce TPS genes account for many of the major and minor terpenoid compounds of the defensive oleoresin and volatile emissions. However, there are several distinct types of TPSs still to be found in spruce based upon the terpenoid components identified in oleoresin. Based on the current phylogeny of functionally characterized spruce TPSs, we predict that most of the remaining TPSs to be identified will be highly similar in sequence to previously identified TPS, but with the possibility of diverse function due to relatively minor sequence divergence.

In contrast to the many duplicated TPS-d genes of terpenoid specialized metabolism, the related spruce TPS genes of general gibberellin phytohormone biosynthesis, specifically *ent*-copalyl diphosphate synthase (TPS-c) and *ent*-kaurene synthase (TPS-e), appear to be expressed as single copy genes [12]. These primary metabolism TPS genes are basal to the specialized metabolism genes and are the descendants of an ancestral plant diterpene synthase similar to the one found in the non-vascular plant *Physcomitrella patens *[12,48]. The mechanisms that suppress manifestation or retention of TPS gene duplication in diterpenoid primary metabolism and those that enhance TPS gene duplication and functional diversification in specialized metabolism in a conifer genome are not known but are worthy of future investigation. The high functional plasticity of the TPS-d family and the great diversity of terpenoids produced may impart fitness advantages against a multitude of pests and pathogens. We speculate that the TPS-d genes of specialized metabolism originating from gene duplication are slower, or less likely, to become inactive pseudogenes compared to those genes with less functional plasticity in primary metabolism.

## Conclusions

Based upon estimates from EST and FLcDNA sequencing in three species of spruce, the TPS gene family in conifers appears to be at least of comparable size to those found in angiosperms with sequenced genomes. This study highlights the great diversity of TPSs of specialized metabolism in conifers, which resulted from gene duplication and functional diversification. Functional differences can occur naturally due to small differences in amino acid sequence.

## Methods

### *In silico *identification of spruce terpene synthases in the EST and FLcDNA databases

Quality trimmed and filtered nucleotide sequences were obtained from spruce genomic resources developed in the Genome Canada-funded Treenomix (http://www.treenomix.ca) and Arborea (http://www.arborea.ulaval.ca) projects as follows: white spruce (242,931 ESTs), Sitka spruce (174,384 ESTs), and hybrid white spruce (also referred to as interior spruce; 26,350 ESTs) [15,16]. Conifer TPS protein sequences available from NCBI were used to query the three species-specific databases using the tBLASTn module of WU-BLAST 2.0 and an E-value cut off of 1 × 10^-5^. The resulting outputs were filtered to exclude duplicates, and then assembled separately by species using CAP3 [49] using an overlap of 40 bp and a percent identity of 95%. The assembled TPS candidate sequences were then tentatively annotated using NCBI BLASTx using the nr database (downloaded Oct. 2008).

### Selection of FLcDNA clones for functional characterization

Authentic cDNA clones corresponding to the above-identified TPS candidate sequences were examined further by restriction digest, colony PCR, and/or sequencing. Those clones that potentially contained a full-length TPS cDNA (i.e. complete ORF) were fully sequenced and if a unique full-ORF TPS was found, the insert was subcloned for expression as described below. In one case, two full ORFs (WS00725_G07c1, WS00725_G07c2) were obtained by 5'-RACE and the full-length genes were subsequently cloned into pCR Blunt II TOPO (Invitrogen).

### Cloning of *PsTPS-Iso*

Because of our particular interest in conifer diTPSs [12,24] and the low abundance of putative diTPSs in the ESTs, we chose to isolate an isopimaradiene synthase (*PsTPS-Iso*) cDNA from Sitka spruce using homology-based cloning to allow functional comparison with its putative levopimaradiene/abietadiene synthase paralog (*PsTPS-LAS*, WS0299_C21, described here). Examination of the spruce EST resources [15,16] identified a 3'-read for clone WS00752_D05 from white spruce with high similarity to the isopimaradiene synthase from Norway spruce (*PaTPS-Iso*; [14]). Full sequencing of this cDNA clone indicated that it was an incomplete transcript. Using PCR with primers designed for the 3'-UTR of this sequence and the 5'-UTR of WS0299_C21, we amplified a 2,700 bp cDNA from the bark of methyl jasmonate-treated Sitka spruce (genotype Haney 898). The amplicon was cloned into pCR Blunt II TOPO and fully sequenced (*PsTPS-Iso*, pSW06061903).

### Expression and purification of recombinant TPS enzymes

TPS cDNAs were amplified using proof-reading polymerase (Phusion, Finnzymes, Espoo, Finland) and subcloned into *Nde*I/*Hin*dIII-digested pET28b(+) (Novagen) using a sticky-end PCR approach [50], or via topoisomerase-mediated insertion into pET100 TOPO/D or pET200 TOPO/D (Invitrogen). All resulting recombinant proteins were full-length and N-terminally His-tagged. Expression constructs were fully sequence verified.

Plasmids were transformed into chemically competent C41 *E. coli *cells (http://www.overexpress.com) containing the pRARE 2 plasmid (coding for rare tRNAs) prepared from Novagen Rosetta 2 cells (EMD Biosciences, Inc., Madison, WI, USA). Luria-Bertani medium (5 mL) containing appropriate antibiotics was inoculated with three individual colonies and cultured overnight at 37°C, 220 rpm. Terrific Broth medium (50 mL) containing appropriate antibiotics was then inoculated with 0.5 mL of the overnight culture and grown in a 250 mL baffle flask at 37°C and 300 rpm until an optical density at 600 nm of at least 0.8 was reached. Cultures were then cooled to 16°C, induced with 0.2 mM IPTG, and then cultured for approximately 16-20 h at 16°C and 220 rpm before pelleting and freezing.

Cell pellets were resuspended, lysed, and sonicated in (1.5 mL g^-1 ^pellet) ice cold 20 mM NaPO_4_, 500 mM NaCl, 30 mM imidazole, 0.04 mg mL^-1 ^DNase, 1 mM MgCl_2_, 5 mM PMSF, and 0.5 mg mL^-1 ^lysozyme, pH 7.4 and then clarified by centrifugation (30 min, 12,000 × *g*, 4°C). The cleared lysates were applied to His SpinTrap Ni-affinity columns (GE Healthcare, Piscataway, NJ, USA) and eluted with 20 mM NaPO_4_, 500 mM NaCl, and 500 mM imidazole, pH 7.4 at 4°C following the manufacturer's protocol. Purified enzymes were desalted at 4°C into 25 mM HEPES pH 7.2, 100 mM KCl, and 10% glycerol using PD MiniTrap G-25 desalting columns (GE Healthcare) and then used immediately for enzyme assays.

### Enzyme assays and gas chromatography-mass spectrometry (GCMS) analyses

Single-vial enzyme assays were completed in triplicate in 2 mL amber glass GC sample vials as previously described [51] in three different buffer/substrate combinations with approximately 60 μg of purified protein per 500 μL assay. Buffers consisted of: monoTPS assays; 25 mM HEPES, pH 7.2, 100 mM KCl, 10 mM MnCl_2_, 5 mM fresh DTT, 10% glycerol, and 51 μM GPP (geranyl diphosphate, Sigma-Aldrich, Oakville, ON); sesquiTPS assays; 25 mM HEPES, pH 7.2, 10 mM MgCl_2_, 5 mM fresh DTT, 10% glycerol, and 43 μM FPP ((*E*,*E*)-farnesyl diphosphate, Sigma-Aldrich); diTPS assays; 50 mM HEPES, pH 7.2, 100 mM KCl, 7.5 mM MgCl_2_, 20 μM MnCl_2_, 5 mM fresh DTT, 5% glycerol, and 37 μM GGPP ((*E*,*E*,*E*)-geranylgeranyl diphosphate, Sigma-Aldrich). Assays were overlaid with 500 μL of pentane and incubated at 30°C for 90 min after which they were vortexed for 30 s to denature the proteins and extract the products into the pentane layer. To completely separate the phases prior to GCMS analysis, samples were frozen at -80°C and then the vials were centrifuged for 30 min at 1,000 × *g *at 4°C.

Assay products were analyzed on an Agilent HP-5ms column (5% phenyl methyl siloxane, 30 m × 250 μm ID, 0.25 μm film) at 1 mL min^-1 ^He on an Agilent 6890N gas chromatograph, 7683B series autosampler (vertical syringe position of 8 to sample the pentane layer), and 5975 Inert XL MS Detector. GC temperature program as follows: 40°C, hold 1 min, 7.5°C min^-1 ^to 250°C, hold 2 min, pulsed splitless injector held at 250°C. Samples were also analyzed on an Agilent DB-WAX column (polyethylene glycol, 30 m × 250 μm ID, 0.25 μm film) with the following temperature program: 40°C, hold 3 min, 10°C min^-1 ^to 240°C, hold 15 min, pulsed splitless injector held at 240°C. Compounds were identified by comparison of mass spectra and retention indices with authentic standards if available, and retention indices, and/or mass spectra from Adams [52] and NIST, and combined mass spectra and retention index library searches in MassFinder [53] if standards were not available.

When possible, stereochemistry of enzyme products were compared to authentic chiral standards on an Agilent Cyclodex B column (permethylated β-cyclodextrin in DB 1701 ((14%-cyanopropyl-phenyl)-methylpolysiloxane), 30 m × 250 μm ID, 0.25 μm film) with the following temperature program: 55°C, hold 1 min, 1°C min^-1 ^to 100°C, 10°C min^-1 ^to 240°C, hold 10 min, pulsed splitless injector held at 230°C.

### Phylogenetic analysis

Protein alignments were prepared using MUSCLE [54] and phylogenetic trees were constructed using the neighbour-joining method with 100 bootstrap repetitions, both within CLC Main Workbench 5.6.1 (CLC bio, Århus, Denmark).

### Molecular modelling

We used Deep View/Swiss-PDBViewer (Mac version 3.9.1b) and SWISS-MODEL [55-57] to develop a 3D homology model of WS00725_G07c1 truncated at Q63 based on the structure of limonene synthase from *Mentha spicata *containing the substrate analogue 2-fluorogeranyl diphosphate (Protein Data Bank 2ONGB) [35].

## List of abbreviations

TPS: terpene synthase; EST: expressed sequence tag; FLcDNA: full-length cDNA; GPP: geranyl diphosphate; FPP: farnesyl diphosphate; GGPP: geranylgeranyl diphosphate; GC: gas chromatography; MS: mass spectrometry; ORF: open reading frame; MeJA: methyl jasmonate; gene and enzyme names abbreviations are shown in Table 3.

## Authors' contributions

CIK, SGR, and JB conceived the research. SGR and SJ selected and sequenced the clones for functional characterization and completed RACE. CIK, SW, BH, and HKD cloned, expressed and/or functionally characterized the clones. CIK and JB wrote the manuscript. All authors read and approved the final manuscript.
